# Social Determinants of Health in the Development of Cardiovascular-kidney-metabolic Syndrome

**DOI:** 10.31083/RCM26580

**Published:** 2025-03-07

**Authors:** Xinyi Cai, Tuo Li

**Affiliations:** ^1^Department of Endocrinology, Changzheng Hospital, Second Military Medical University, 200003 Shanghai, China

**Keywords:** social determinants of health (SDOH), cardiovascular-kidney-metabolic (CKM) syndrome, screening, prevention, management

## Abstract

Cardiovascular-kidney-metabolic (CKM) syndrome is characterized by the interactions among the metabolic risk factors, chronic kidney diseases (CKD) and cardiovascular diseases (CVD). Social determinants of health (SDOH) include society, economy, environment, community and psychological factors, which correspond with cardiovascular and kidney events of the CKM population. SDOH are integral components throughout the entire spectrum of CKM, acting as key contributors from initial preventative measures to ongoing management, as well as in the formulation of health policies and the conduct of research, serving as vital instruments in the pursuit of health equity and the improvement of health standards. This article summarizes the important role of SDOH in CKM syndrome and explores the prospects of comprehensive management based on SDOH. It is hoped that these insights will offer valuable contributions to improving CKM-related issues and enhancing health standards.

## 1. Background

Cardiovascular-kidney-metabolic (CKM) syndrome is a worldwide public health issue that impacts millions of people, resulting in substantial healthcare and economic burdens. Currently, social determinants of health (SDOH), encompassing several factors associated with societal, financial, environmental, and mental aspects, have a significant impact on health status and healthcare accessibility. When it comes to the relationship between SDOH and CKM, studies have shown that SDOH not only affect the onset and progression of CKM but also play a crucial role in its screening and management [1, 2]. Screening for SDOH is beneficial for the early detection of CKM risk factors and timely medical intervention. Despite the increasing awareness of the importance of SDOH in health outcomes, a cohesive framework for SDOH screening is still lacking, and the impact of SDOH interventions on CKM has not yet been supported by comprehensive data and research. In the study by Brandt *et al*. [3], SDOH is considered a major driver of adverse cardiovascular outcomes, and approaches to address SDOH were summarized in the context of health care providers and health systems. However, addressing SDOH necessitates sustained financial and communal support, which is currently impeded by limited public awareness and underdeveloped policies. Moreover, disparities in the distribution of healthcare resources, a disjointed healthcare system, and the lack of multidisciplinary collaboration also contribute to adverse SDOH. This review seeks to delve into the influence of SDOH on CKM screening, prevention, and management, and discuss strategies for addressing SDOH from the combined efforts of government, community, and healthcare systems, with the goal of improving CKM health and providing valuable references for the promotion of health equity.

## 2. A Main Overview of CKM 

CKM was proposed by the American Heart Association (AHA) in a presidential advisory, defined as a systemic disorder attributable to interactions among metabolic risk factors, chronic kidney diseases (CKD) and cardiovascular diseases (CVD), leading to multiorgan dysfunction and a high rate of adverse cardiovascular outcomes [4].

Diabetes is a global pandemic, impacting 500 million lives worldwide, disproportionately in low and middle-income populations and countries [5]. Diabetes increases all-cause mortality largely from cardiovascular and renal diseases and contributes to multiple complications, including blindness, limb loss, chronic pain, and disability [6, 7]. There is a growing recognition that cardiovascular diseases and kidney diseases are also closely linked through shared biological and social risk factors, known as cardiorenal syndrome [8, 9, 10]. The pathophysiological association among dysfunction of metabolism, CKD and CVD is complex, the core processes of which are inflammation, oxidative stress, vascular dysfunction and insulin resistance [11, 12]. In the presidential advisory, the AHA further classifies CKM into stages ranging from 0 to 4, taking into account the patients’ metabolic risk factors, renal function, and cardiovascular health. This allows for the development of personalized management and intervention strategies for individuals with different cardiorenal risks, to mitigate cardiovascular risks and the progression of renal impairment. Since it is a multidisciplinary disease, the management of CKM requires a comprehensive approach, including lifestyle modifications, pharmacological therapies, and regular monitoring of cardiovascular and renal function.

## 3. Cardiovascular Health and SDOH

In 2022, the AHA issued an advisory, introducing an updated and enhanced approach, namely, Life’s Essential 8, to measuring, monitoring, and promoting ongoing efforts to improve cardiovascular health (CVH) in all populations [13]. The 8 components of the new CVH definition are divided into two domains of health behaviors (diet, physical activity, nicotine exposure, sleep) and health factors (body mass index, blood lipids, blood glucose, blood pressure). Acting as interacting gears among the continuous interplay of brain-mind-heart-body connections, the eight metrics can lead to bidirectional effects in CVH. A modified Delphi approach is utilized to add up the scores scaled from 0 to 100 points, informed by health outcomes and risk associations. The aggregate scale for assessment of CVH is calculated as the unweighted average scores of the eight components.

SDOH was conceptualized within the Essential 8 structure, defined as the structural determinants and conditions in the environment where people are born, grow, live, work, and age that affect health, functioning, and quality of life outcomes and risks [14]. These circumstances are shaped by the distribution of money, power, and resources at global, national, and local levels. The social determinants of health are mostly responsible for health inequities—the unfair and avoidable differences in health status seen within and between nations [15].

There are four commonly referenced consensuses for the classifications and terminology of SDOH: the World Health Organization (WHO) Commission on Social Determinants of Health [16], Healthy People 2020 [14], the County Health Rankings Model [17, 18], and Kaiser Family Foundation (KFF) Social Determinants of Health factors [19, 20]. The KFF SDOH framework, which is widely recognized, categorizes social risks into six domains: economic stability, neighborhood and physical environment, education, food insecurity, social and community context, and the healthcare system. Each domain encompasses a detailed compilation of specific factors.

**Fig. 1.**
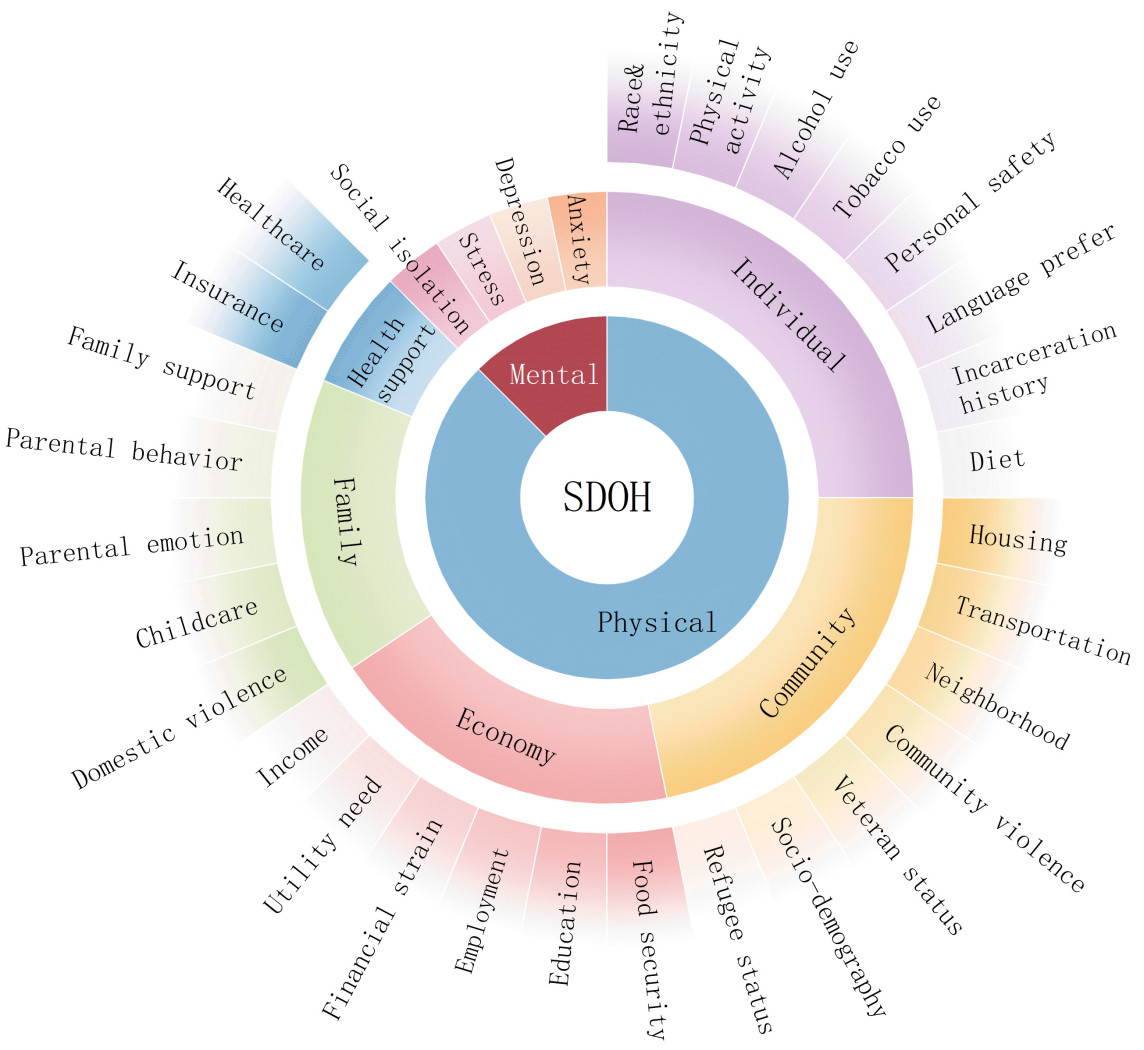
**Framework of social determinants of health (SDOH)**. SDOH is divided into mental and physical domains. The physical domain breaks down into five specific types: economy (income, utility need, financial resource strain, employment, education, and food security), health support (insurance coverage and healthcare), community (housing instability, transportation challenges, neighborhood, community violence, veteran status, refugee status, and socio-demographic information), family (domestic violence, childcare, parental emotion, parental behavior, and family support), and individual (race and ethnicity, physical activity, alcohol use, tobacco use, personal safety, language preference, incarceration history, and diet). The mental domain includes social isolation, depression, stress, and anxiety. Utility needs indicates difficulty paying utility bills, shut off notices, lack of access to a phone; financial resource strains include the inability to afford essential needs, financial literacy, medication under-use due to cost, and benefits denial; transportation challenges, difficulty accessing or affording transportation (medical or public); socio-demographic information such as race and ethnicity, educational attainment, family income level, and languages of the community; childcare including preschool, after-school programs, prenatal support services, kids clothing and supplies, summer programs; social isolation includes lack of family and/or friend networks, minimal community contacts, absence of social engagement.

Nonetheless, based on existing screening instruments, the article comes up with a novel classification framework for SDOH (Fig. 1). It begins by distinguishing two broad categories: physical and mental domains. The physical domain is further divided into five subcategories: economic factors, health support systems, community attributes, familial elements, and personal lifestyles. Each of the five subcategories encompasses subordinate classifications, along with four subcategories in the mental health domain, amounting to a comprehensive list of 32 distinct items.

## 4. Relationship between SDOH and CKM

The increased incidence of CKM syndrome and its adverse outcomes is further influenced by unfavorable conditions for lifestyle and self-care resulting from policies, economics and the environment. For individuals with adverse SDOH, the combination of metabolic disorders, CVD and CKD which comprise CKM syndrome offers a better view of its prevention and management based on CKM staging.

The interplay between SDOH and the progression of CKM syndrome is multifaceted, influenced by various factors including genetics, behavior, and environment conditions. An adverse burden of SDOH increases the likelihood of progression in the CKM staging. SDOH is connected with disparities in cardiovascular health behaviors, and poor SDOH at both the individual and community levels impact cardiovascular risk and mortality. Moreover, SDOH play an important role in the progression, diagnosis, and outcomes of CKD and type 2 diabetes (T2D), and correlates with an increased risk of renal failure.

## 5. Screening of SDOH for the Detection of CKM

Screening for SDOH among patient groups is essential for both the prevention and management of the CKM syndrome. A range of approaches, such as questionnaires, face-to-face interviews, community assessments, and data analysis, can be utilized to conduct SDOH screening. The results of such screenings can guide personalized healthcare strategies and the development of public health initiatives tailored to the needs of specific communities. In this way, SDOH screening contributes to achieving more equitable and effective health care delivery and resource allocation.

**Fig. 2.**
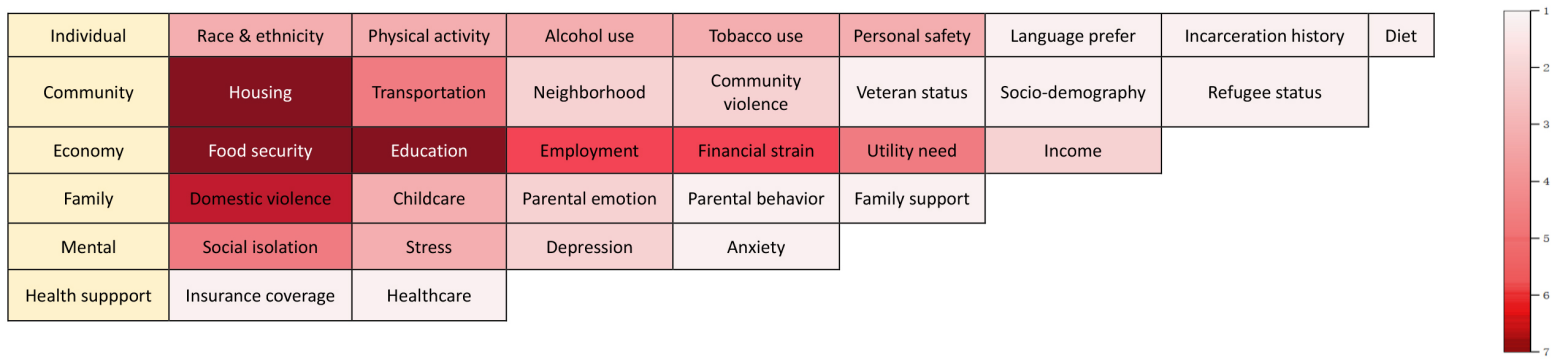
**Heat map for SDOH screening**. This heatmap illustrates the occurrence of specific screening items in current SDOH assessment tools. The color intensity of each rectangle corresponds to the quantity of tools that encompass this item, with the color scale escalating from 1 to 7. A deeper color indicates a greater number of tools including the item, while a lighter color demonstrates that the item is present in fewer assessment tools. The information is sourced from a comprehensive analysis of current SDOH screening instruments.

Numerous screening tools have been developed to evaluate SDOH, including Health Leads [21], American Academy of Family Physicians (AAFP) [22], Protocol for Responding to and Assessing Patients’ Assets, Risks and Experiences (PRAPARE) [23], Oregon Community Health Information Network (OCHIN) [24], Safe Environment for Every Kid Parent Screening Questionnaire (SEEK PSQ) [25], Income, Housing, Education, Literacy, and Personal Safety (IHELP) [26], and the Well Child Care, Evaluation, Community Resources, Advocacy, Referral, Education (WE CARE) [27]. The Centers for Medicare & Medicaid Services (CMS) developed a screening instrument consisting of 10 items to detect medical requirements that can be solved in communities [28]. This article analyzes the eight tools and categorizes them into six groups comprising 32 screening items, and demonstrates the occurrence of these items through a heatmap (Fig. 2). Most available screening instruments include the measurement of mental health to assess depression, social isolation, or stress. Health Leads and PRAPARE have been developed with steps for incorporation into the clinical care workflow, and the former can specifically address health literacy. SEEK PSQ, WE CARE and IHELP were developed to screen caregivers for SDOH among pediatric populations.

## 6. Application of SDOH in CKM

SDOH are pivotal in the prevention and management of the CKM syndrome. They shape the health behaviors, conditions, and outcomes at both individual and population levels. Enhancing the well-being of CKM patients requires a multifaceted approach with various strategies and interventions. Below are some concrete ways in which SDOH contribute to the prevention and management of CKM.

### 6.1 Economic Stability

Economic stability is composed of employment, income, job opportunity, social welfare, poverty, and parent socioeconomic status in children and adolescents.

Financial constraints influence an individual’s capacity to obtain healthy foods, medical care, exercise facilities, and engage in a healthy lifestyle. Therefore, individuals with poor finances are more likely to develop cardio-renal metabolic conditions, including cardiovascular diseases, diabetes, and obesity [29]. The higher the income and the higher occupational grade, the less engagement in physical activity [30], the less likely that individuals develop obesity, T2D, CVD or to experience their complications. Inequalities in mortality and CVD outcomes exist in China as well, whose indicators involves education, occupation, and household wealth and a composite socioeconomic-status disparity index [31]. Thus, improving economic conditions can improve the risks associated with CKM syndrome.

The CMS has developed the health-related social needs (HRSN) screening tool to help healthcare and social service providers to screen for economic stability [32]. Clinicians can use the HRSN screening tool to assess the economic stability of their patients. Individuals who are identified through the HRSN tool as requiring support may be directed to suitable programs offered by healthcare entities or community resources. Clinicians can also ask patients about employment status or whether their income meets basic needs for food, shelter, and health. If there are issues with jobs or money, patients can be linked to social welfare programs for the necessary assistance.

The healthcare system should be positioned to serve as a partner in addressing issues concerning economic stability. It is advised to offer financial support and job opportunities to low-income CKM patients to ensure their capability of paying healthcare costs such as medications, treatments, and medical devices, allowing them to gain access to better healthcare services and healthy food options. Furthermore, initiatives like social welfare programs, including retirement funds, unemployment relief, disability assistance, and food subsidy, can be implemented to lighten the financial burden on CKM patients and improve the quality of their diet.

### 6.2 Social and Community Context

Including an early childhood family system involves family support, social network support, social activity participation, stressful life events, stigma and discrimination, exposure to violence and/or trauma, and involvement in the criminal justice system.

A systemic review of 18 observational studies of adults with T2D found that higher levels of social support were associated with improved outcomes, including better glycemic control, treatment adherence, quality of life, diagnosis awareness and acceptance, and stress reduction [33].

Given the limited power of an individual, impactful mitigation strategies should be implemented through policy and regulatory measures to prioritize the vulnerable and underserved populations. In society and community, a variety of measures can be taken for the prevention and management of CKM syndrome. Neighborhoods may initiate health education programs to advocate for healthy lifestyles and enhance public awareness of CKM diseases and their risk factors. Health screenings should be regularly organized to promptly identify CKM-related conditions [34]. For individuals with CKM, community-based support systems can be established to offer health counseling and emotional support, which also assist in understanding and securing community resources.

Additionally, community research can be conducted to understand the prevalence and determinants of CKM diseases in specific communities. Through these measures, society and local communities can foster a supportive environment for the self-management of CKM patients and reduce the risks of CKM-related diseases.

### 6.3 Neighborhood and Physical Environment

The neighborhood and physical environment includes built environment, physical activity, housing stability, transportation, and food security.

#### 6.3.1 Built Environment

It has been demonstrated that the built environment is associated with obesity. Twohig-Bennett and Jones [35] conducted a meta-analysis to examine the relationship between diabetes outcomes and “high” and “low” exposure to greenspace in neighborhoods. The analysis included 462,220 participants, and demonstrated the correlation of higher exposure with lower incidence of T2D [35].

Communities should make more effort in their environment, including the expansion of green areas, improvement of air quality, and the establishment of secure areas for physical activity. Additionally, they may develop public spaces like parks, plazas, and community centers for leisure and social communication, all of which contribute to the prevention of CKM.

#### 6.3.2 Physical Activity

It is well recognized that physical activity (PA) is of significant importance to enhancing physical fitness, improving cardiac reserve, and promoting blood circulation [36, 37]. With the development of technology and the progression of transportation tools, PA has declined notably in modern society. Instead, sitting for a long time has become the daily routine for many people, which is closely linked to higher risks of diabetes, hyperlipidemia, hypertension, and CVD. Therefore, regular exercise can help promote health and mitigate the risk factors of cardiometabolic diseases [38, 39, 40]. Adults are advised to undertake 150 minutes of moderate-intensity or 75 minutes of vigorous aerobic PA weekly, along with strength training exercises at least twice a week [41].

The healthcare system can contribute to promoting PA in patients through a multifaceted strategy. This includes a thorough evaluation of levels of PA intensity and screening for various obstacles that may prevent individuals from meeting PA goals. Referrals can then be coordinated to the care specialists. For example, exercise therapists, can assess physical constraints and make up personalized PA plans. Additionally, nutritionists may also be of help in providing dietary guidance and recommendations to fit personal physical requirements. In this way, the healthcare system can address gaps in knowledge and foster PA practices over time.

#### 6.3.3 Housing Instability, Transportation and Food Insecurity

Housing instability is a worsening social issue that can influence overall physical and mental health [42]. Studies have demonstrated that both the risks of developing CVD and T2D are higher in individuals facing unaffordable housing [43, 44]. Among adults in unstable housing, CVD is the major cause of death, with a CVD mortality of 2 to 3 times higher than the general population [45, 46].

Access to transportation is an essential component of health care [47]. For elderly people with limited mobility and people living in urban-rural and rural areas, limited transportation prevents them from accessing timely and effective medical services. Transportation barriers may also affect the frequency of seeking medical care such as clinic visits, which is a disadvantage for the diagnosis and management of patients with chronic diseases. In contrast, convenient transportation is crucial to ensure timely medical visits and treatment, which further determine the prognosis and even the survival rate. Therefore, for acute complications of the CKM syndrome, such as acute myocardial infarction (AMI), atrial fibrillation, stroke, acute onset of CKD, and diabetic ketoacidosis, easy access to transportation is of great importance [48].

Food security is defined as access by all people at all times to enough food for an active and healthy life. Nutrition security is defined as a condition of having equitable and stable availability, access, affordability and utilization of foods that are beneficial to health and prevent and treat diseases. Food insecurity in the community and at individual levels is associated with an increased incidence and prevalence of diabetes, and poor access to food leads to worse long-term outcomes. Furthermore, lack of stable access to food can also contribute to an elevated risk of metabolic related diseases except for diabetes, including obesity, hypertension, and dyslipidemia, which in turn can promote the progression of CKM syndrome [49, 50].

To solve the issues related to housing, transportation and food security, the government can formulate relevant policies to improve housing and transportation conditions, and to promise reliable access to food supplies. This will contribute to the early prevention, diagnosis and management of CKM syndrome.

### 6.4 Education

Education encompasses education attainment, early childhood health education and school-based support, race/ethnic segregation in schools, and vocational training.

From the perspective of disease prevention and diagnosis, people with lower levels of education tend to have lower health literacy, meaning their awareness of disease prevention and health risks is comparatively limited. This may lead to numerous unhealthy behaviors in their daily lives, such as smoking, drinking, imbalanced diet, and an irregular daily routine. At the same time, due to an insufficient emphasis on health, they may lack regular health check-ups, which could affect the early diagnosis of many chronic diseases. A lower level of education is related to an increased risk of AMI, coronary heart diseases, stroke, heart failure, sudden cardiac death, and all-cause mortality [29, 51]. During the medical consultation process, individuals with higher levels of education are often able to communicate more effectively with doctors and understand their advice. This is particularly important for chronic diseases such as diabetes, obesity, and CKD, as patients need to take medicine and monitor long-term health indicators, which demands compliance and self-care skills.

Given the association between education level and health literacy, increasing the rate of high school completion is a key step in advancing health equity, which requires the joint efforts of communities and governments [52]. At the community level, health education should be strengthened to raise the overall health awareness among the public. For instance, people should be informed that obesity and conditions such as hypertension and hyperlipidemia are early stages of the CKM. When individuals discover these risk factors, they should promptly adjust their lifestyle and visit a hospital for primary prevention and early intervention to reduce the risk of developing the CKM. In the diagnostic and treatment process, clinicians can use open-ended questions and simple language when talking with patients who have lower levels of education, in order to communicate more effectively, thereby enabling timely diagnosis and treatment.

### 6.5 Healthcare System

The healthcare system is composed of insurance coverage, provider and pharmacy availability, access to care, and quality of care.

In contemporary medical practice, the use of internet connectivity and telehealth solutions is becoming more prevalent in the long-term management of chronic conditions. Through remote monitoring tools, physicians can continuously monitor the health indicators of patients with CKM syndrome, including blood glucose levels, blood lipid levels, blood pressure, and kidney function. This enables prompt virtual consultations and interventions, ensuring effective care and the creation of tailored treatment plans for each patient [53]. Telehealth facilitates the delivery of medical services directly to patients’ homes or local community centers, minimizing the physical and temporal demands of hospital visits and reducing the need for clinic visits and hospitalization, which can eventually lead to a decrease in overall healthcare expenses [54, 55]. It is especially beneficial for individuals in remote areas, providing them with access to quality healthcare services that were previously inaccessible. In this way, the gap between urban and rural medical services can be narrowed, which further promotes healthcare equity [56, 57].

Being a complex disease, a multidisciplinary approach is even more necessary in the management of CKM. Telemedicine platforms enable collaboration between specialists from various fields, such as cardiology, nephrology, and endocrinology, to co-manage patients and offer more holistic diagnostic and treatment services. Additionally, telemedicine offers a means of aggregating substantial datasets from a vast array of patient data, which help to study the epidemiology and therapeutic outcomes of CKM. The insights gained from this data can inform and enhance strategies for the prevention, management, and ongoing care of CKM in the future.

The management of CKM can be considered from the following aspects.

#### 6.5.1 Insurance Coverage

Expanding health insurance coverage to ensure that CKM patients have access to necessary medical services and medications. In addition, implementing insurance programs specifically designed for the ongoing management of chronic diseases to lessen the financial burden on patients [58].

#### 6.5.2 Provider and Pharmacy Availability

Governments and insurance companies can negotiate with pharmaceutical manufacturers to reduce drug prices. For low-income patients, implement medication assistance programs to provide them with free or low-cost drugs [59].

Hospitals may lower the expense of drugs through consolidated purchasing or volume buying, which take advantage of the cost benefits associated with large-scale orders. Promoting the use of generic drugs as an alternative to expensive patented medications, which typically offer the same therapeutic benefits at a lower price.

#### 6.5.3 Access to Care

Offering telehealth services enables CKM patients in geographically isolated areas to receive professional medical monitoring, consultation, and treatment remotely.

#### 6.5.4 Quality of Care

Clinicians should possess a high level of professional integrity and, by working in a multidisciplinary team, offer holistic and integrated medical care for patients with CKM. Additionally, electronic health records and data analysis tools can be utilized to monitor the quality and effectiveness of healthcare services, offering timely feedback and improvement measures [60].

In summary, SDOH exert an impact on the risks of CKM syndrome across multiple factors. Therefore, during the formulation of public health policies and clinical intervention measures, these social factors should be taken into account to promote more comprehensive healthcare and the management of CKM.

## 7. Current Deficiencies and Prospects

While the significance of SDOH is broadly acknowledged, there remains several gaps and hurdles in their implementation and study, yet this also presents numerous potential approaches for exploration and advancement.

### 7.1 Lack of Screening Tools

There is a lack of systematic and comprehensive SDOH screening tools for early detection and diagnosis of CKM syndrome. To address the gaps in CKM research, it is necessary to incorporate SDOH assessment protocols and data into the framework of electronic health records and clinical workflows.

### 7.2 Evaluation and Monitoring 

The current landscape is marked by an absence of potent assessment instruments capable of the evaluation of the influence and efficacy of interventions targeting SDOH. The design of cross-sectional studies restricts insights into the progression of trends over time [61]. Subsequent longitudinal research should evaluate the effects of interventions on CKM outcomes. Health outcomes and healthcare expenditure should also be measured to identify interventions that are economically viable. Moreover, further research is essential for deepening the understanding of the interplay between SDOH and the CKM, and for developing mechanisms to assess the efficacy of SDOH interventions in CKM management.

### 7.3 Research

Current research commonly focuses on identifying optimal approaches for the assessment and quantification of SDOH in individual and community settings. The understanding of the best methods to measure and quantify SDOH is currently limited, and the key determinants for maintaining CVH across individuals and populations remain to be elucidated. Future studies should broaden the spectrum of SDOH to fill the voids related to community resources, social support, and environmental factors that have not yet been sufficiently explored, thereby offering a more holistic evaluation [62].

### 7.4 Public Awareness and Policy Making

The public awareness of SDOH is insufficient, which restricts societal support for health promotion policies and contributes to health inequities. To transcend the conceptual boundaries of CKM syndrome, it is essential to enhance health education and raise citizens’ awareness of preventive screening and early diagnosis [63]. On a societal level, community workers should enhance health advocacy to boost the understanding of SDOH, encourage preventative actions, and facilitate early intervention. It is the responsibility of governments to establish impactful policies aimed at addressing SDOH, including promoting economic conditions, housing standards, educational opportunities, and healthcare equity, and increase funding and resource allocation for research studies related to SDOH and CKM, thereby addressing the long-standing social burdens within the healthcare system.

### 7.5 Interdisciplinary Collaboration

The study and implementation of SDOH necessitate collaborative efforts from multiple fields, yet the current frameworks and processes for such interdisciplinary partnerships are not sufficiently developed. Treating CVD, CKD, and metabolic disorders separately is complex and costly, especially for patients with adverse SDOH. It is essential for healthcare systems to bolster interdisciplinary collaboration among cardiologists, nephrologists, and endocrinologists, and to incorporate considerations of SDOH into the clinical care workflow for CKM.

### 7.6 Collaborative Efforts

In order to enhance the prevention and management of the CKM population, partnerships should be promoted among healthcare providers, community organizations, and government agencies to integrate SDOH into comprehensive strategies for the management of the CKM syndrome, thereby making collaborative efforts to reduce the burden of CKM and enhance health outcomes across all populations.

## 8. Conclusions

SDOH plays a crucial role in the prevention and management of the cardiovascular-kidney-metabolic syndrome. In the context of CKM, the impact of SDOH is particularly significant as these factors affect not only individuals’ health behaviors but also their access to health resources and medical services. Effectively combating CKM syndrome requires a multifaceted, coordinated, and patient-centered approach from government, community, and healthcare systems. By identifying and improving these social determinants, the prevention and management of CKM syndrome can be more efficaciously achieved, health inequities can be diminished, and quality of life can be significantly improved. Tackling CKM based on SDOH is a long-term task that demands the collective dedication and action of all involved.

## Abbreviations

AAFP, American Academy of Family Physicians; AHA, American Heart Association; AMI, acute myocardial infarction; CKD, chronic kidney disease; CKM, cardiovascular-kidney-metabolic; CMS, Centers for Medicare & Medicaid Services; CVD, cardiovascular diseases; CVH, cardiovascular health; HRSN, health-related social needs; IHELP, Income, Housing, Education, Literacy, and Personal Safety; KFF, Kaiser Family Foundation; OCHIN, Oregon Community Health Information Network; PA, physical activities; PRAPARE, Protocol for Responding to and Assessing Patients’ Assets, Risks and Experiences; T2D, type 2 diabetes; SDOH, social determinants of health; SEEK PSQ, Safe Environment for Every Kid Parent Screening Questionnaire; WE CARE, Well Child Care, Evaluation, Community Resources, Advocacy, Referral, Education; WHO, World Health Organization.
